# Exploring the Links between Obesity and Psoriasis: A Comprehensive Review

**DOI:** 10.3390/ijms23147499

**Published:** 2022-07-06

**Authors:** Gabriela Barros, Pablo Duran, Ivana Vera, Valmore Bermúdez

**Affiliations:** 1 Departamento de Post-Grado, Universidad Católica de Cuenca, Ciudad Cuenca 010109, Ecuador; ana.barros.23@est.ucacue.edu.ec; 2 Endocrine and Metabolic Diseases Research Center, School of Medicine, The University of Zulia, Maracaibo 4004, Venezuela; pabloduran1998@gmail.com (P.D.); ivanaa19.iv@gmail.com (I.V.); 3 Facultad de Ciencias de la Salud, Universidad Simón Bolívar, Barranquilla 080002, Colombia

**Keywords:** adipokines, cytokines, inflammation, obesity, psoriasis, microbiota, body mass index

## Abstract

Obesity is a major public health issue worldwide since it is associated with the development of chronic comorbidities such as type 2 diabetes, dyslipidemias, atherosclerosis, some cancer forms and skin diseases, including psoriasis. Scientific evidence has indicated that the possible link between obesity and psoriasis may be multifactorial, highlighting dietary habits, lifestyle, certain genetic factors and the microbiome as leading factors in the progress of both pathologies because they are associated with a chronic pro-inflammatory state. Thus, inflammation management in obesity is a plausible target for psoriasis, not only because of the sick adipose tissue secretome profile but also due to the relationship of obesity with the rest of the immune derangements associated with psoriasis initiation and maintenance. Hence, this review will provide a general and molecular overview of the relationship between both pathologies and present recent therapeutic advances in treating this problem.

## 1. Introduction

Obesity is a highly prevalent, chronic, and multifactorial endocrine-metabolic disease characterised by an excessive increase in body weight due to abnormal accumulation of body fat [1]. In this regard, the Metabolically Unhealthy Obese phenotype (MUO) is associated with cardiovascular [2,3,4], osteoarticular [5], hepatobiliary [6], psychological [7], neurological [8], and immune system diseases [9], besides some forms of cancer [10]. In this context, obesity is considered a public health issue of significant scale and a challenge for healthcare systems considering its complex management and the economic impact of its comorbidities [11,12]. If secular trends continue, it is estimated that by 2030, 38% of the world’s adult population will be overweight, and 20% will be obese [13]. In the USA, dire predictions indicate that more than 85% of adults will be overweight (overweight + obese) by 2030 [14]. Although the trend in increasing obesity prevalence appears to have been stabilised in some countries (particularly in Europe), morbid obesity rates continue to increase, as do the cases in many developing countries [15].

Currently, it is not up for debate that the fundamental cause of obesity lies in an energy imbalance due to increased calorie intake coupled with a physical activity decrease [14]. Therefore, eating habits and physical activity changes are a consequence of environmental and social changes associated with development [16,17], transculturation and industrialisation [15], as well as the lack of policies supporting health [10,18], agriculture [16,19], transportation, urban planning [16], environmental [10,20], food processing, distribution/marketing [20,21] and education sectors [14,18].

As alluded previously, obesity plays an essential role in developing multiple diseases, many of which are caused by insulin resistance due to increased fatty acid synthesis and release from visceral adipose tissue to organs such as the liver and skeletal muscle [14]. In this context, substantial evidence has been accumulating regarding an emerging hypothesis that complements the well-known classical view of the adipose-insular axis. It is based on alterations in the composition and function of the microbiota [22] (intestinal, upper respiratory tract and skin) as a driver for the development of chronic systemic inflammation characteristic of the obesity-diabetes-metabolic syndrome continuum [4,23,24,25]. This fact reveals a more complex system, the Microbiota-immune-adipose-neuroendocrine axis, on which alterations such as beta-cell apoptosis [26], fatty acid liver disease (and subsequent liver cirrhosis) [27], neuroinflammation with cognitive impairment [28], dysfunctional adipose tissue [29] (due to hypoxia and Endoplasmic Reticulum stress), accelerated atherosclerosis [30] gravitate around inflammation and autoimmunity [3].

Obesity plays a fundamental role in skin conditions. It represents a risk factor for several skin pathologies, including acanthosis nigricans, acne, hyperhidrosis, intertriginous dermatitis and acrochordons [31]. This correlation has been associated with insulin resistance and compensatory hyperinsulinemia, where the innate [32] and adaptive immune system [33,34,35], pro-inflammatory cytokines and adipokines are responsible for triggering chronic inflammation [36,37]. Such events could, at least in theory, alter the skin physiology in terms of synthesis and structure of collagen [38], sebaceous glands function, sweat glands, and skin layer’s maturation [38,39].

During the natural history of obesity, adipocytes become senescent and dysfunctional, shifting their proteomic programming toward a pro-inflammatory phenotype that may play a fundamental role in the immune system function. In genetically susceptible individuals, this could represent the battleground where many skin pathologies could develop [40]. Thus, plantar hyperkeratosis, cellulitis, keratosis pilaris, *striae distensae*, hidradenitis suppurativa and skin infections have been associated with obesity [38]. Furthermore, regarding other diseases, including rosacea and psoriasis, evidence has been growing that obesity is a risk factor for their onset [41,42,43,44].

In this context, a recent meta-analysis by Ko et al., which evaluated ten randomised controlled trials with interventions of at least 12 weeks involving 1163 participants, highlighted the possible link between skin diseases such as psoriasis and obesity. This study yielded two findings of interest: firstly, the psoriasis prevalence increased as body mass index (BMI) [44] augmented, and secondly, a relationship was found between psoriasis severity and BMI [44]. Furthermore, as discussed below, an important finding in many other studies is that clinical manifestations such as psoriatic flares improve with weight reduction and physical activity [1,21,39,42,45], highlighting the importance of the obese phenotype study in the management and prognosis of psoriasis [36,46].

Therefore, this review aims to study the possible links between obesity and psoriasis from an epidemiological, immuno-molecular, clinical and therapeutic perspective and provide explicitly and synthesised scientific information on the link between these pathologies. 

## 2. Materials and Methods

This review provides novel information on the link between obesity and psoriasis; the literature review was not systematic. An extensive search was performed on Scopus, EMBASE, PubMed, ISI Web of Science, ScienceDirect, Medline and Cochrane Library Plus databases from inception to April 2022. The articles recovered for this review were only those in English. No restrictions were made according to study type, and only scientific articles from high-impact journals were selected (Q1, Q2 and Q3). The terms “Obesity”, “Psoriasis”, “Obesity and Psoriasis”, “Chronic inflammation and psoriasis”, and “Microbiota and Psoriasis” were the main keywords used throughout the search.

## 3. The Link between Psoriasis and Obesity

### 3.1. Is There Epidemiological Evidence That the Increase in the Prevalence of Obesity Coincides with the Epidemiological Behaviour of Psoriasis over the Last 50 Years?

The analysis of the possible role of obesity in the development of psoriasis is of utmost importance since it is to be expected that as the prevalence of obesity may worsen (a phenomenon that occurs in many countries), we should also be prepared to identify a more significant number of psoriasis cases [47] and its multiple comorbidities [40,45,48].

Globally, psoriasis affects 2 out of every 100 people [40,49], with a current prevalence of 125 million people [21,31], 7.5 million people in the United States [50], and 6 million people in China [40], making it a global health issue and a significant economic burden [51]. However, the prevalence in Eastern countries is lower than in Western countries, e.g., Taiwan with 0.24%, Japan with 0.34%, China with 0.47% and Korea with 0.54% prevalence of psoriasis [34]. Likewise, in these oriental countries, obesity prevalence is significantly lower, ranging from 4 to 7% [17]. Nevertheless, an essential fact in psoriasis epidemiology is the lack of information about its incidence since 81% of the countries do not report data [52,53]. Despite this, according to 2010 to 2017 [54] statistics, world psoriasis prevalence is around 2%, affecting more frequently countries with higher economic income [52]. Furthermore, a cohort study of incidence conducted by Icen et al., followed for 30 years from 1970 to 2000, showed a significant two-fold increase in psoriasis incidence with a male sex predominance; however, women over 60 years of age had a greater predisposition [53]. On the other hand, in some Middle East, North Africa and Oceania countries, the prevalence of weight excess (obesity + overweight) has surpassed the 50% of the adult population [55], while obesity prevalence in European countries is about 20% [55]. In this regard, a systematic review and meta-analysis of observational studies conducted by Xie W et al., including 16 studies with a sample of 322,967 people, showed that the risk of psoriatic arthritis was higher in people who were obese and overweight with an OR of 1.75 (95% CI, 1.42–2.16) and OR 1.50 (95% CI, 1.08–2.09) respectively; escalating 6% for each kg/m^2^ increase in body mass index [56].

### 3.2. Relationship between Anthropometric Indices and Psoriasis

Several studies have evidence that psoriasis risk rises alongside BMI increasing [25,57,58] (Table 1). For example, a retrospective study conducted by Norden et al. in the United States of America (USA) with a sample of 1.5 million patients followed for 11 years showed a statistically significant difference in psoriasis frequency across the different BMI categories. Therefore, the prevalence of psoriasis was 9.5% in patients with normal weight, 11.9% in overweight patients, 14.2% in grade I obesity and 17.4% in patients with grades II–III obesity [58]. Similarly, this increase in body mass index, which leads to obesity, is related to metabolic syndrome diagnosis, a significant risk factor for Psoriasis development [1,4,34]. In this context, it is vital to note that some studies have found a higher Psoriasis treatment failure rate in patients with obesity [49,59]. In addition, several experts have highlighted that the bidirectional relationship between obesity and psoriasis starts from a pro-inflammatory state coupled with excessive adipokines secretion such as leptin [60], a phenomenon shared by these pathologies [33,43,50,61].

A prospective longitudinal study conducted by Setty et al., in a sample of 78,626 women followed for 14 years with controls every two years, confirmed an increase in the incidence of psoriasis regarding BMI, waist circumference and hip circumference, with statistically significant *p*-values, as evidenced by 892 cases of psoriasis in people who presented an increase in these anthropometric measures. The multivariate relative risks of psoriasis were 1.40 (95% CI, 1.13–1.73) for a BMI of 25.0–29.9; 1.48 (95% CI, 1.15–1.91) for a BMI of 30.0–34.9; and 2.69 (95% CI, 2.12–3.40) for a BMI of 35.0 or greater (*p* = 0.001). For BMI above 30 kg/m^2^ it was 1.73 (95% CI, 1.24–2.41) and for BMI below 21 kg/m^2^ it was 0.76 (95% CI, 0.65–0.90) (*p* = 0.001) [62]. Furthermore, a proof-of-concept and open-label clinical trial conducted by Castaldo et al. observed that a 12% weight reduction in patients with obesity resulted in a PASI decrease of 50–75%, with significant improvement in psoriasis severity and quality of life index score [48].

### 3.3. Food and Psoriasis: Is There a Link?

**Figure 1:**
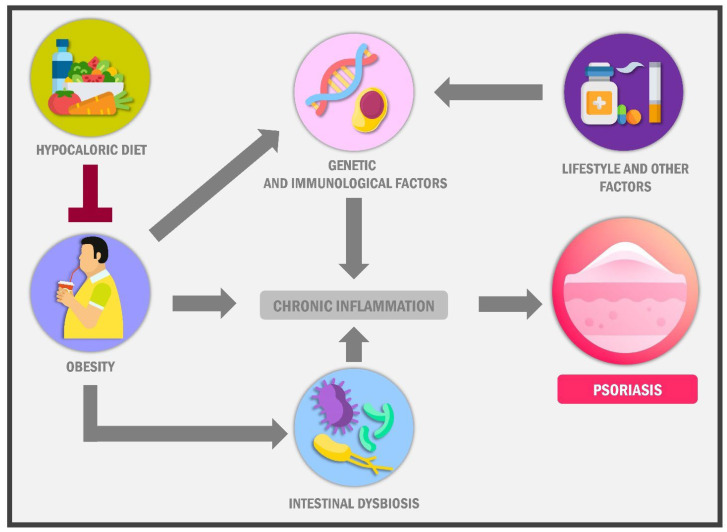
Biological factors involved in psoriasis pathophysiology. Obesity has been suggested as one of the mainstay factors of chronic inflammatory processes associated with psoriasis. Furthermore, it has been described how adipokines and pro-inflammatory cytokines contribute to psoriasis development from the sick adipose tissue. Obesity and a hypercaloric high-fat diet are linked to intestinal dysbiosis, leading to decreased intraluminal short-chain fatty acid production and local inflammation caused by the proliferation of “non-friendly” bacteria at gut epithelium leading to mucosal damage and increased permeability of gut mucosa. The damage to the gut mucosal layer subsequently causes pro-inflammatory cytokines release, leading to systemic inflammation. In turn, lifestyle factors and obesity alter gene expression and, thus, deregulation in critical immune cell functions like T-reg lymphocytes, Macrophages, and dendritic cells, exacerbating the inflammatory response. The persistence in this chronic not-solved systemic process plays a critical role in psoriasis pathogenesis.

In the Americas, increased consumption of ultra-processed foods and fast foods known as the “Western diet” has been adopted, leading to overweight and obesity prevalence explosion and, in turn, all its comorbidities [17]. This diet provides significant calorie amounts and is low in nutritional quality, consisting of foods rich in saturated fats, sugar, and sodium [63]; this contributes to increased body weight and metabolic diseases. Therefore, bodyweight reduction through a hypocaloric diet decreases visceral adipose tissue, Leptin, IL-6 and IL-1a [21] while increasing ketone bodies, attenuating inflammatory response linked to psoriasis and obesity [32] (Figure 1). Thus, the multiple changes in the dietary patterns in Eastern and Western societies are influenced by household income, food prices [20,21], religious beliefs, geographical aspects [17], food preferences [16] and traditions [15], amongst others [21], which could modify the interplay between obesity and psoriasis worldwide.

The cardiometabolic risk in people with obesity is often observed in patients with severe psoriasis too [50]. Obesity, hyperlipidemia and type 2 diabetes mellitus are associated with moderate and severe psoriasis, while non-alcoholic fatty liver disease is more prevalent in obese patients with psoriasis [64] than those without these conditions [27,45]; however, psoriasis alone can lead to liver fibrosis [27]. From a clinical point of view, psoriasis comorbidities such as cardiovascular disease, type 2 diabetes mellitus, dyslipidemia, obesity and metabolic syndrome are highly relevant since they reduce life expectancy, especially when the condition is severe [49].

New strategies to support psoriatic patient management are now developed from the epidemiological link between obesity-nutrition-psoriasis as modifiable risk factors [39]. Weight loss through an adequate diet with a low caloric index has been linked to inflammation decrease at intestinal, skin and cardiometabolic levels. Additionally, a decrease in C-reactive protein, TNF-α and IL-6 have been observed, achieved by soluble diet fibre consumption [3].

### 3.4. Role of the Gut and Skin Microbiota

A key component in the pathogenesis of psoriasis and obesity is the microbiota [29,63], which is altered [61], sharing this characteristic with other chronic inflammatory diseases. The microbiota comprises all microorganisms that habitually inhabit the internal and external surfaces of the human body. They are strongly related to autoimmune diseases [65,66], where there is an increased production of IL-17 [33] and an imbalance in lymphocyte production [67]. The microbiota is responsible for protecting and maintaining skin balance at the skin level by forming an immune barrier against harmful external agents [61]. Bacterial translocation and leaky gut syndrome support the state of chronic systemic inflammation and trigger the onset of psoriasis [67], obesity, certain cardiovascular diseases and inflammatory bowel diseases [67].

In obesity, significant alterations have been observed in the microbiota characterised by changes in bacteria composition regarding Firmicutes and Bacteroidetes *ratio* compared to normal-weight individuals [68]. Likewise, obesity-related differences have been described regarding the content of microbes such as *Clostridium innocuum*, *Eubacterium dolichum*, *Catenibacterium mitsuokai*, *Lactobacillus reuteri*, *Lactobacillus sakei*, and *Actinobacteria*, as well as in rarer *Archaea* organisms like *Methanobrevibacter smithii* [69].

Therefore, dysbiosis, a key characteristic of obesity, indicates an intimate correlation between both elements, as demonstrated in a comparative study by Turnbaugh et al., in which 16S rRNA gene sequences from the distal microbiota of obese mice showed an increased Bacteroidetes to Firmicutes ratio. However, upon further investigation, it has been established that dysbiosis is not the only factor influencing obesity since the relationship between both is much more complex, where elements including short-chain fatty acids synthesis, lipopolysaccharides release and pro-inflammatory cytokines, among others, have a significant impact [70,71].

In this sense, a study by Lv et al. explored the association between gut microbiota and BMI in obese and lean Chinese male university students. This work showed that microbiota diversity decreased as BMI increased, while the faecal sample’s composition from normal-weight individuals was more complex [72]. Similarly, an exploratory study conducted by Barengolts et al. studied the association between microbiota biomarkers and their influence on obesity in a cohort of African-American men consisting of 41 obese and 34 normal-weight persons as a reference. Biomarkers like short-chain fatty acids (SCFAs), the percentage of lipopolysaccharide-binding protein (LBP) and the ratio of LBP to CD14 (LBP/CD14) were higher in the obese (n = 41, age 58 years, BMI 36 kg/m^2^) than in normal-weight men (n = 34, age 55 years, BMI 26 kg/m^2^). BMI correlated significantly with LBP, LBP/CD14 (*p* < 0.05 for both) and SCFAs (propionic, butyric, isovaleric, *p* < 0.01 for all). These studies show the influence of microbiota composition and related factors in the development of obesity [73].

Although dysbiosis mechanisms are not fully understood, it has been hypothesised that during obesity, a diet that is poor in soluble fibre and rich in saturated fatty acids like palmitic acid changes the gut microbiota architecture, which results in bacterial-driven digestion of the protective mucus layer [74]. Similarly, changes in the microbiota in obesity decrease the expression of a lipoprotein lipase inhibitor called fasting-induced adipose factor (FIAF); consequently, there is an increase in fat storage [69].

On the other hand, the predominance of gram-negative bacteria as a result of dysbiosis in obesity could alter the protein structure of the intestinal cell junctions, increasing the permeability of the intestinal surface as well as bacterial lipopolysaccharides (LPS) absorption leading to pro-inflammatory cytokines such as IL-6, tumour necrosis factor-alpha and C-reactive protein release, increasing the inflammatory process during obesity development [75]. In addition, endogenous cannabinoid system activation by microbiota by endothelial CBI receptors overstimulation. As a result, e intestinal barrier function is compromised and, therefore, the levels of LPS in plasma [76].

Regarding psoriasis, as occur with obesity, the dysfunctional gut microbiota has been reported [22]. Numerous studies have consistently found an increase in *Corynebacterium kroppenstedtii*, *Corynebacterium simulans*, *Neisseria* spp., *Finegoldia* spp., *Faecalibacterium genus*, *Salmonella* spp., *Campylobacter* sp., *Helicobacter* sp., *Escherichia coli*, *Alcaligenes* sp. and *Mycobacterium* sp. On the other hand, a decrease in *Cutibacterium, Burkholderia* spp., *Bacteroidetes phylum*, *F. prausnitzii*, *Bifidobacterium* spp., *Lactobacillus* spp., *Parabacteroides* and *Coprobacillus* [68]. These pathological changes in microbiota support the chronic inflammatory state hypothesis [29] based on lipoteichoic acid and lipopolysaccharides released into the bloodstream [39]. Furthermore, metabolic enzyme imbalances in iron transport, cobalamin, and carbohydrates are commonly exhibited by patients with psoriasis due to altered gut microbiota [77]. Hence, some clinical studies suggest the clinical manifestations of psoriasis can be significantly improved or ameliorated with the improvement of the gut microbiota [22,63,78]. A cohort study conducted by Hidalgo et al. in which faecal microbiota from 19 patients with psoriasis and 20 without psoriasis belonging to the same geographic location was evaluated through 16S rRNA gene sequencing with total DNA and bioinformatic analysis found a clear dysbiosis in psoriatic patients with a lower microbiota diversity and a differential abundance of specific bacterial species [63].

These facts open new avenues in the relationship between the gut microbiota disturbances in obesity and the initial triggers in keratinocytes activation like Adypokines, IL-6 and TNF-α from the sick adipose tissue and the LPS pass from a leaky gut due to advanced dysbiosis, and finally, innovative therapeutic approach in psoriasis [63].

### 3.5. A High-Fat Diet and Palmitic Acid Consumption Break the Gut Epithelium Integrity and Initiate Pro-Inflammatory Cytokine Production

The subclinical systemic inflammation observed during obesity has been attributed to the immune response to increased circulating levels of LPS from the cell wall of gram-negative bacteria, termed metabolic endotoxemia. The passage of bacterial fragments into the blood through the intestinal mucosa emphasises the importance of the intestinal epithelial barrier in this process [79]. However, some studies have begun to deepen this paradigm. For example, in a recent study conducted by Genser et al., In a cohort of 122 obese and non-obese patients, the intestinal barrier function was analysed by combining in vivo and ex vivo investigations, finding alterations of the tight junctions in the jejunal epithelium of obese patients, evidenced by a reduction of occludin and tricellulin [80].

Similarly, serum levels of zonulin and LPS-binding protein, two markers associated with alterations of the intestinal barrier, were also increased in obese patients, as well as augmented jejunal permeability to small molecules (0.4 kDa) related to systemic inflammation in the obese cohort, proving, overall, that intestinal barrier function is subtly compromised in obese patients. However, the most exciting data of this study were related to the administration of a saturated fatty acid load, finding that jejunal permeability after lipid loading was twice as high in obese patients compared to non-obese controls and correlated with systemic and intestinal inflammation, evidenced by an increase in IL-6 and IL-1B production [80].

In conclusion, alongside gut dysbiosis, another two early local phenomena could behave as triggers of psoriasis in patients with obesity: the early intestinal inflammatory response to a high-fat diet, especially palmitic acid, and the subsequent impairment in intestinal permeability with passage into the circulatory stream of bacterial lipopolysaccharides that together can activate and polarise monocytes to M1 macrophages, activating dendritic cells and even keratinocytes [81,82].

### 3.6. Obesity and Endoplasmic Reticulum Stress in Adipocytes Increases Pro-Inflammatory Cytokine Production in Adipose Tissue

The second focus of inflammatory immune activation and a significant source of palmitic acid is the expanding and dysfunctional adipose tissue observed in obesity [83]. Adipocytes are professional cells for storing lipids in the form of triacylglycerides, molecules with a high energy density that outside adipose tissue tend to be toxic. Anatomical location and genetic factors largely determine the proportional increase in the size and number of adipocytes and their physiological responses. Nevertheless, as obesity progresses, preadipocyte differentiation becomes dysfunctional, leading to reduced insulin signalling, glucose uptake and adiponectin release by mature adipocytes. This process is due to at least two phenomena: Firstly, the growth and expansion of adipose tissue due to hypertrophy restrict oxygen diffusion from the capillaries to the adipocytes, producing hypoxia [84]. The second, palmitic acid from the high-fat diet or lipolysis, drives endoplasmic reticulum (ER) stress and unfolded protein response (UPR) activation [85,86].

The UPR is activated in response to unfolded or misfolded proteins and its further accumulation in the endoplasmic reticulum lumen. In this scenario, UPR has three objectives: initially to restore normal cell function by halting protein translation, degrading misfolded proteins, and activating signalling pathways that lead to increased production of molecular chaperones involved in protein folding. If these targets are not achieved, or the interruption in protein synthesis is prolonged, the UPR targets apoptosis [87].

The UPR is constituted by three metabolic pathways that are initiated by the activation of specific ER transmembrane proteins such as protein kinase RNA-like ER kinase (PERK), Inositol Requiring Enzyme-1 (IRE1) and Activating Transcription Factor 6 (ATF6). On the other hand, the light of the ER is replete with special chaperones such as the immunoglobulin heavy chain binding protein (BiP), also called glucose-regulated protein 78 (GRP78). Under normal conditions, all these stress sensors remain inactive by forming a complex with BiP/GRP78. However, the aggregation of misfolded and unfolded proteins within the ER lumen causes BiP/GRP78 and rapid activation of the three ER stress receptors, generating a cell survival signal. Nevertheless, if this signal is prolonged, the global response will be preferentially pro-apoptotic [88].

PERK is a protein kinase that phosphorylates downstream proteins, such as eukaryotic initiation factor-2α (eIF2α), resulting in an overall reduction of protein synthesis and consequently a decrease in the misfolded/mutant proteins load in the ER. However, this blockade is incomplete, so several mRNAs, including the one encoding the ATF transcription factor, are still translated, leading to the expression of proteins such as GADD34 and the transcription factor homologous protein C/EBP or CHOP. Interestingly, this pathway through transcription factor CHOP is known to be pro-apoptotic via the death receptor 5. However, the mRNA of this receptor is cleaved by IRE1α endonuclease, which promotes CHOP, at first, to exhibit pro-inflammatory properties by repressing the expression of three essential genes: (1) the adiponectin gene, (2) the Eotaxin gene and the IL-13 gene, producing peripheral insulin resistance, decreased IL-4 production and decreased IL-13 signalling, favouring macrophage polarisation to M1 [89,90]. This circle is closed by high Leptin levels acting in an autocrine and paracrine fashion on adipocytes, activating the mTOR pathway, which induces α-TNF and CCL- 2 synthesis (chemokine C-C motif ligand 2) in neighbouring adipocytes attracting monocytes to the adipose tissue, promoting a pro-inflammatory milieu that rapidly polarises to M1 macrophages [91]. This set of immunometabolic alterations is amplified and perpetuated by activating the endoplasmic reticulum stress pathway IRE-1. 

Thus, stimuli such as hypoxia and palmitic acid generate a highly pro-inflammatory environment via dysfunctional adipocytes and M1 macrophages within adipose tissue. These pro-inflammatory molecules (IL-1b, IL-6, TNF-a, Leptin, IL-12, IL-23) pass into the bloodstream stimulating inflammation in multiple tissues, including the skin, and represent a biologically plausible explanation for the relationship between obesity and psoriasis both in its onset and perpetuation [92,93].

### 3.7. Palmitic Acid Directly Affects the Activation of Antigen-Presenting Cells and Keratinocytes

It is well described that, in general, exogenous saturated fatty acids (ESFA) exert pro-inflammatory effects on dendritic cells (DC) and macrophages. Interestingly, several recent studies have revealed an essential role for metabolic program change in this process. It was shown that activation of ESFA in Acyl-CoA activates the NLPR3 inflammasome, driving to an M1 polarisation, whereas polyunsaturated fatty acids (PUFAs) prevented it [94]. These authors demonstrated that ESFA promoted phosphatidylcholine synthesis, leading to membrane fluidity and K+ efflux loss, allowing NLRP3 activation. PUFAs were able to inhibit this effect by redirecting SFA to triacylglycerol synthesis.

Furthermore, macrophage exposure to palmitic acid was associated with chronic low-grade inflammation and increased synthesis of IL-1β and IL-23 [95]. This phenomenon was related to hexokinase inhibition by palmitic acid and Krebs cycle disruption in TLR-activated cells, leading to increased mitochondrial ROS production and pro-inflammatory cytokines. However, the exact mechanisms or receptors by which ESFA modulate macrophages or DC function have not been fully elucidated yet, but it has been observed that ESFA could bind to TLRs, thereby activating a pro-inflammatory phenotype in macrophages [96,97].

The first work suggesting that saturated ESFA have an activating effect on keratinocytes came from Zhou et al. These investigators treated HaCaT keratinocytes with palmitic acid, observing that IL-6, TNF-*α*, IL-1*β*NF-, NF-*κ*B were increased by palmitic acid. In contrast, this effect was attenuated by blocking NF-*κ*B pathway with pyrrolidine dithiocarbamate (PDTC, a selective chemical inhibitor of NF-*κ*B) [82].

### 3.8. Typical High-Fat Diet (Palmitic Acid) of the Westernised Countries Produces Smooth Endoplasmic Reticulum Stress in Multiple Tissues, including Keratinocytes

As explained, many metabolic pathways and immune system cells are activated—in the absence of pathogens, by aberrant metabolism during adipose tissue expansion and dysfunction. This intimate relationship between ER stress and inflammation is involved in restoring cellular homeostasis and leads to metabolic diseases. Therefore, Zhao et al., studied ER stress in keratinocytes from normal skin and psoriasis Vulgaris lesions. They found that the ER of people with psoriasis was ultra-structurally abnormal. In contrast, western blotting and immunostaining techniques showed that proteins such as BIP and CHOP were abnormal compared to healthy people [98].

A recent study conducted by Nakamizo et al. in mice with imiquimod-induced psoriasis and high-fat diet-induced obesity discovered a new mechanism leading to the exacerbation of psoriasis lesions due to IL-17 production via T-cells γδ in the skin and lymph nodes. These results and other previous studies suggest the potential role of fatty acids in overexpressing CCL20 mRNA in keratinocytes. It has been reported that palmitic acid facilitated IL-17-induced keratinocyte activation in vitro and that there is a strong correlation between the amount of palmitic or oleic acid in serum and the severity of skin inflammation in HFD-induced exacerbation of psoriatic dermatitis. Thus, both saturated and monounsaturated fatty acids seem to be involved in the effect of HFD on psoriatic dermatitis [99]. For example, Vasseur et al. reported an increase in IL-17A-producing cells in the skin and an exacerbation of IMQ-induced psoriatic dermatitis in mice [100] when a high-fat diet containing saturated fatty acids was administered. In addition, prolonged exposure of primary epidermal keratinocytes to TNF-α decreases cathepsins levels in lysosomes with the consequent reduction in autophagy, a finding also observed in patients with psoriasis suggesting that psoriasis-associated cytokines, such as TNF-α and IL-17A, alter autophagy in keratinocytes [101].

### 3.9. Alteration in the Inflammation Resolution in Psoriasis

**Figure 2:**
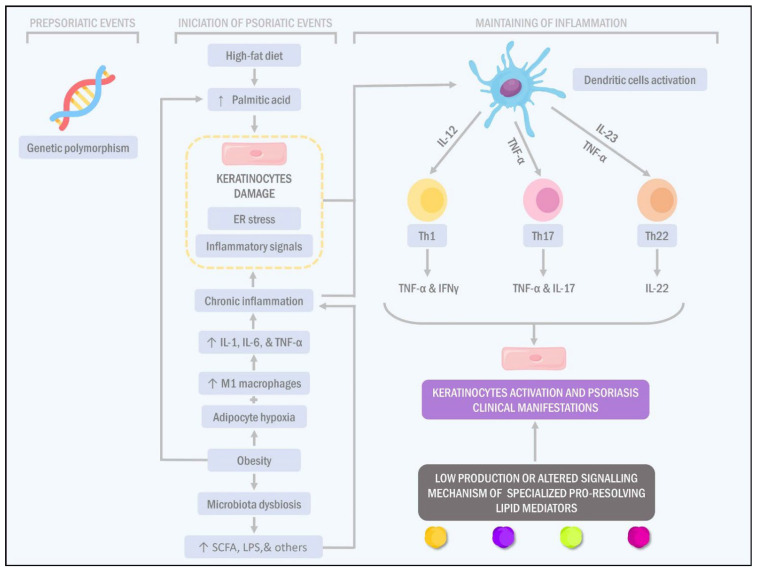
Role of obesity, high-fat diets, and inflammation in the pathophysiology of psoriasis. Psoriasis development has three stages: prior to the processes involved in the disease pathogenesis, the onset of psoriatic events and the maintenance of inflammation associated with psoriasis. Concerning the first stage, genetic polymorphisms play a crucial role. Regarding the onset of the psoriatic events, high-fat diets and obesity may increase palmitic acid levels, which is related to endoplasmic reticulum stress in keratinocytes and adipocytes, leading to a change in their secretory activity and, therefore, the establishment of inflammatory processes. In turn, obesity contributes to establishing a state of hypoxia in the adipose tissue that aggravates local inflammation, which, together with the activation of M1 macrophages, exacerbates the systemic inflammatory state. Obesity and high-fat diets are also associated with intestinal dysbiosis, which may contribute to increased intestinal permeability that allows the passage of pro-inflammatory toxic substances and immune system modulators into the bloodstream. Together, the aforementioned inflammatory processes cause damage to epithelial tissue and keratinocytes. In addition, these mechanisms are capable of activating cells of the immune lineage, such as dendritic cells and T-helper lymphocytes (Th1, Th17 and Th22). Systemic inflammation and exacerbated activation of keratinocytes cause the appearance of the psoriasis lesions. It is worth mentioning that, typically, these mechanisms tend to be resolved by the presence of inflammatory mediators such as specialised pro-resolving lipid mediators; however, their production and signalling mechanisms are altered under these conditions.

The initiation and resolution of inflammatory responses are governed by the sequential activation, migration, control and/or suppression of immune cells in which bioactive lipids play an essential role in regulating these two processes in a dynamic and timely manner (Figure 2).

Mediators derived from omega-6, omega-3 fatty acids and arachidonic acid (AA) play a crucial role in inflammation and its resolution and are fundamental in the inflammatory cascade initiation. AA is an omega-6 polyunsaturated fatty acid (PUFA) that leads to the production of eicosanoids. The biosynthesis of these mediators depends on the activity of cyclooxygenases (COX-1, COX-2) or lipoxygenases (5-LOX, 12-LOX, 15-LOX). These eicosanoids include prostaglandins (PG), thromboxanes (TX) and leukotrienes (LT). Inhibition of COX-2 prostanoid production by acetylsalicylic acid (ASA) changes the enzymatic activity of an endoperoxide synthase, leading to endogenous aspirin-activated lipid mediators (AT) production, including anti-inflammatory and pro-resolving lipoxins [102,103].

These lipid signalling are involved in various pathophysiological processes by modulating appropriate inflammatory responses to various stimuli and the environment [104]. The inflammatory response is usually a self-limiting process that, under normal conditions, should conclude with inflammation resolution by the involvement of a series of lipid messengers derived from Omega-3 polyunsaturated fatty acids like eicosapentaenoic acid (EPA) and docosahexaenoic acid (DHA) leading to the specialised pro-resolving lipid mediators (SPMs) biosynthesis. These bioactive mediators, which include resolvins (Rv), protectins (PD) and maresins (MaR), have anti-inflammatory and immunomodulatory actions in many animal models and represent significant potential for treating human inflammatory diseases where failed resolution evidenced by aberrant production of SPM or its precursors is already recognised as a key pathophysiological mechanism in their genesis. Thus, an altered SPM/eicosanoid ratio in peripheral blood/tissue predicts disease progression and treatment efficacy [105,106].

Numerous clinical trials have been conducted on psoriasis with Omega-3 fatty acids. However, the limited number of study participants, insufficient concentration or purity of omega-3 supplements, the time of administration, and the application of low-sensitivity clinical diagnostic tools have resulted in conflicting, albeit promising, results. Furthermore, it should be noted that none of these studies has determined possible changes in the lipidomic profile of psoriatic lesions. The first study that controlled the presence of PMS in individuals with psoriasis was recently presented by Sorokin et al., who identified a set of AA- and SPM-derived prostanoids in the skin and peripheral blood of patients with psoriasis. This study found, like other works, that AA-derived prostanoids were significantly increased in the skin with psoriasis lesions and plasma of these patients, especially PGE2 [107]. It should be remembered that PGE2 is a critical pro-inflammatory mediator responsible for hyperalgesia, which stimulates the production of pro-inflammatory cytokines. On the other hand, at least in theory should reactivate the resolution and biosynthesis programs of SPM-lipoxins, Rvs, PD and maresins within inflammatory exudates [108].

Interestingly, this study reported that lipoxins LXA4 and LXB4 were near the lower detection limits in the skin and peripheral blood samples with a trend toward the elevation of LTB4 and its isomer, 5S, 12S-diHETE in psoriasis patients. In this regard, LTB4 is a potent neutrophil chemoattractant that also increases the motility of dendritic cells in the skin, which induces reorganisation of actin filaments and causes increased skin inflammation. This imbalance in lipoxin-leukotriene homeostasis could explain, at least in part, the abrogated inflammation resolution profile observed in psoriasis [107].

### 3.10. Is Maresin-1 the Missing Link in the Resolution of Psoriasis Inflammation?

Recently, Maresin’s role in inflammation resolution failure in psoriasis has been highlighted because Maresin-1 enhances phagocytic activity in macrophages promoting their anti-inflammatory action by M2 polarisation (CD11c- CD206+) as well as inhibiting the polarisation of CD11c+ CD206- (M1) macrophages [109]. Furthermore, it should be remembered that M2 macrophages secrete anti-inflammatory cytokines such as IL-10 and TGF-β, which accelerate tissue remodelling and the elimination of apoptotic debris by phagocytosis, inhibiting the inflammatory response [110]. Likewise, this mediator has been shown to suppress the production of IL-1β. In addition, maresin-1 inhibits IL-6 and TNF-α production by suppressing the SIRT1/PGC-1α/PPARγ pathway [111].

Recent studies have shown that maresin-1 has anti-inflammatory effects on T cells, essential elements in the cascade of inflammatory events in psoriasis. For example, Maresin-1 can suppress the induction of CD4+ cells, CD8+ cells and Th17 cells by down-regulating T-bet and Rorc expression [112]. In contrast, maresin-1 enhanced the induction of Tregs and the production of the anti-inflammatory cytokine IL-10. Furthermore, maresin-1 negatively regulates IL-23 receptor expression on Tγδ cells through down-regulation of RORγ and IL-23 receptor internalisation. Therefore, maresin-1 suppressed effector cell induction and induced Treg expansion in conjunction with anti-inflammatory cytokine production in T cells [112,113].

Maresin-1 also has remarkable effects on neutrophils and thus on psoriasis. Maresin-1 has been shown to suppress neutrophil infiltration and decrease the production of CXCL1, a major chemokine for recruiting this cell type. In addition, maresin-1 promote neutrophil apoptosis to induce resolution of the inflammatory response [109,114]. Indeed, topical application of maresin-1 showed anti-inflammatory effects in an imiquimod-induced psoriasis mouse model by inhibiting the production of IL-17A by γδTCR mid+ and CD4+ cells in the skin through repression in IL-23 receptor expression. IL-17A production by γδTCR mid+ and CD4+ cells in the skin causes IL-23 receptor expression inhibition [115]. Therefore, topical maresin-1 looks promising for IL-17-related diseases. 

### 3.11. Immunomolecular and Genetic Factors: The Missing Link between Psoriasis and Obesity?

Psoriasis can occur in people with no family history of the disease, but having a parent with an increase in 10 per cent psoriasis risk. Moreover, if both parents have psoriasis, the risk increases by 50 per cent [44,116]. The genetic factor governing psoriasis is explained by the disease’s presence in offspring and described in siblings [117]. In one-third of psoriatic patients, first- and second-degree relatives have a higher incidence of developing Psoriasis Vulgaris; in turn, for a child born from parents with psoriasis, the risk is 50%, while this risk decreases to 8% if only one sibling has psoriasis [118].

In psoriasis, nine loci are susceptible at the chromosomal level; however, it has been observed that environmental factors can trigger this process [118]. The possible genomic regions linked to Psoriasis are PSORS1, PSORS233, PSORS8, and PSORS4, all of which have an epidermal expression in the pathology [118]. In addition, other genes predispose to psoriasis but are directly linked to inflammation, such as interleukins IL-1, IL-6, IL-8 [62], IL-12B, IL-22, IL-23A, IL-23R, IL-2, IL-21, IL-17 [39], vascular endothelial growth factor (VEGF) and interferon-γ25, many of which presenting an altered expression in obesity too.

Antigen-presenting cells produce IL-23, supporting the development of CD4+ memory T cells responsible for the inflammatory state in psoriasis [116,118,119,120], secreting IL-17 [62]. T helper cells, mast cells and neutrophils produce IL-17, which triggers the production of cytokines and chemokines in keratinocytes through an anticipant inflammatory response [39]. This process is accompanied by angiogenesis [2,121] and occurs at the level of the mainly visceral white adipose tissue that functions as an endocrine organ [40]. In psoriasis, the direct pathogenic cells are epidermal keratinocytes, inflammatory T cells, antigen-presenting cells, and dysregulation in the innate and adaptive immune system [33]. This process leads to increased differentiation and proliferation of epidermal keratinocytes, resulting in the typical clinical portrait of psoriasis Vulgaris [118]. The former genetic and immunological aspect is essential because targeted treatment acts at this level. In addition, dendritic cells such as Langerhans cells play a role in psoriasis. They are found in the epidermis and therefore identify, capture and present antigens to T cells at the lymph nodes [118]. Therefore, Langerhans cells are sentinels of the skin’s immune system [118], while B-cell-mediated immunity is an additional phenomenon beyond its direct involvement [122] because B cells have protective functions in many inflammatory processes and infectious diseases [120].

### 3.12. Role of Adipokines in Obesity and Psoriasis

Adiponectin is an essential adipokine in obesity and psoriasis since it promotes fatty acid oxidation and improves insulin sensitivity. Phosphatidylinositol-3 kinase/Akt (PI3K/AKT) activation pathway by adiponectin increases nitric oxide production, acting as a protective factor against atherosclerosis. In addition, this hormone elicits an anti-inflammatory effect by decreasing vascular inflammation, inhibiting pro-inflammatory cytokines like TNF-α, IL-6, IL-1 and IL-17, and increasing IL-10, an anti-inflammatory cytokine [39]. Furthermore, this adipokine is decreased in both psoriasis and obesity [123]; thus, it has been noticed that when a patient’s weight decreases, adiponectin concentrations rise, and their clinical psoriasis manifestations improve [40].

Leptin is another adipokine from the white adipose tissue whose primary function is to regulate the body’s energy balance [41,46], acting on the hypothalamus and producing long-term satiety signals [60]. This secretion depends on adipose tissue accumulation and adipocyte size [121]. In addition, leptin decreases hepatic lipogenesis and increases lipolysis, improving energy expenditure [40]. However, in obesity, leptin resistance is accompanied by hyperleptinemia; the latter is a feature shared with psoriasis [39]. This fact may explain why leptin levels have consistently increased in obese patients and patients with psoriasis, and its elevation correlates directly with PASI score and BMI [41,123]. From an immune point of view, leptin is responsible for increasing pro-inflammatory cytokine production, causing the proliferation of keratinocytes [41,46]. In addition, leptin can affect dendritic and T helper cells responsible for immunity response in psoriasis [121].

Finally, resistin is an adipokine and a potential biomarker in psoriasis pathogenesis and obesity [48]. A meta-analysis of observational studies conducted by Kyriakou et al. in Caucasian and Asian persons found a direct relationship between blood resistin and the severity of psoriasis [39]. Furthermore, a systematic review and meta-analysis of intervention studies, led by Kyriakou et al. in 2018, indicated that resistin concentrations dropped significantly after psoriasis systemic drug therapy [35].

### 3.13. Is Psoriasis Clinic Related to Obesity?

Psoriasis pathogenesis is mostly early-onset, i.e., before the forties, but it can also occur after this age [116]. Its clinical manifestations are multiple, and many of them worsen with obesity [44], having as a central component the immune response [32]. The more common clinical picture of psoriasis is a well-circumscribed erythematous plaque covered with white or silvery scales, which can be large or small [40], thick or thin [118]. These lesions affect extensor surfaces such as elbows, knees, and the navel, lumbosacral region and scalp [117], representing three-quarters of all psoriatic patients [39], commonly known as chronic plaque psoriasis [31,32,47] or Psoriasis Vulgaris. Other but less frequent types of psoriasis are inverse Psoriasis, palmoplantar Psoriasis, erythrodermic Psoriasis, guttate Psoriasis and pustular psoriasis [39]. Mild forms account for two-thirds of all patients with psoriasis, and only one-third are affected with severe forms [124]. The course of psoriasis usually relapses, but it can sometimes become permanent and severe, considering there is no cure [44]. Psoriatic arthritis is seronegative inflammatory arthritis [29], in which a critical risk factor for its occurrence is the presence of obesity [125]. Psoriasis may occur before, simultaneously, or after arthritis has occurred or never appeared; it may be polyarthritis, oligoarthritis, arthritis *mutilans* or spondylitis [126]; and it affects a quarter of all cases of psoriasis [127].

An important feature shared by psoriasis and obesity is the psychosocial impact and the concomitant reduction in quality of life [22,31]; owing to social isolation, wildly when these two pathologies coexist in the same patient [128], due to a synergistic interaction leading to a more significant deterioration of psychological stability and overall health status [119]. Therefore, the psychological health evaluation is mandatory when assessing treatment response [22] since anxiety or depression strongly predisposes to treatment failure [118]. Moreover, A type of psoriasis in which a genetic association is not described, such as guttate psoriasis, is directly associated with environmental factors like infections and stressful situations [33]. As obesity plays a vital role in the onset and severity of psoriasis, it must be addressed through weight loss to improve the clinical response to nutritional, psychological and pharmacological interventions [35,65,129].

Despite having a genetic factor that explains the manifestation of psoriasis, it is necessary to know that its expression also depends on the interaction with multiple environmental risk factors, such as viral and bacterial infections, obesity, consumption of drugs [58], tobacco and ethyl alcohol consumption; the latter two cause an increment in TNF-α converting enzyme levels, increasing the risk of psoriasis [44].

### 3.14. Psoriasis Treatment Considerations in Obese Patients

It is of utmost importance to recognise all the clinical aspects of the obese patient with psoriasis since it entails a series of metabolic complications [130,131], representing an evident difficulty in making therapeutic decisions, especially when it comes to systemic pharmacological therapy [45]. Since as mentioned above, the drugs used at high doses increase the adverse effects rate, mainly a rise in cardiometabolic complications. Obese patients suffer more significant complications due to adverse effects of antipsoriatic drugs [34] because, in obese patients, higher doses are required [1,25]. It is not unusual to observe that pharmacological response is much better in patients with normal weight [45,132].

Methotrexate, cyclosporine and acitretin are drugs commonly used in the treatment of psoriasis; acitretin has a longer-lasting effect, and the effect of cyclosporine on lesions is faster, while methotrexate and acitretin have a similar effect [133]. However, these drugs can cause non-alcoholic fatty liver disease and liver fibrosis in obese patients when administered at high doses [45]. The psoriasis course is improved by drugs suppressing inflammatory pathways [91] and stimulating regulatory T cells. These goals can be achieved—at least in part—by consuming polyunsaturated fatty acids, short-chain fatty acids [92], vitamin D [86], vitamin B12, probiotics, dietary fibres, genistein and selenium [3]. Indeed, Vitamin D analogues are included in the treatment regimen for psoriasis [92] in adjunction to keratolytic drugs and topical corticosteroids [31,86].

Together with all this, biologic drugs have been the most disruptive approach to psoriasis management [131,134]. It is essential to highlight that a pleiad of clinical studies have shown that biological drugs approved to treat psoriasis and psoriatic arthritis are very effective. Moreover, a biologic may offer the most effective treatment for many people with moderate-to-severe psoriasis or psoriatic arthritis. These drugs target four different immune signalling pathways: (1) TNF-α inhibitors [116], such as adalimumab, certolizumab pegol, etanercept, infliximab and golimumab (2). IL-17 inhibitors, such as brodalumab, ixekizumab, and secukinumab. (3) IL-23 inhibitors, like guselkumab, risankizumab, tildrakizumab, and mirikizumab [31,134], and (4) The dual IL-23 and IL-12 inhibitor ustekinumab [119,130,135]. These drugs improve the clinical manifestations of psoriasis in patients with obesity and improve the evolution of psoriatic arthritis [134].

Nevertheless, the BMI increase significantly undermines drug response. Thus, anti-Ils [45] are more sensitive to weight gain and higher fat depots than anti-TNFs [90]. In this way, infliximab and ustekinumab, dosed according to body weight, are usually administered at high doses in obese patients.

### 3.15. New Hope on the Horizon: Microbiota Manipulation and Polyphenols in the Obesity-Psoriasis Intervention

Gut microbiota manipulation, either by a selective introduction of specific live organisms as probiotics or by promoting healthy microorganism growth through non-digestible carbohydrates administration as a form of prebiotics, has opened the door to a future full of new alternatives in the management of a pleiad for immune-related conditions centred in non-resolved inflammation. Current evidence suggests that some systemic diseases can be modulated by altering the cutaneous and gut microbiome [136]. Further understanding of the role of this ecosystem in psoriasis and obesity interplay could lead to new therapies still to be developed. Probiotics have been demonstrated to improve psoriasis by modulating the two main parameters of gut microbiota: Alpha and Beta diversity [137], putting down roots in the quasi-causal role of dysbiosis in the psoriasis pathogenesis in people with obesity. Furthermore, several by-products from gut microbiota metabolism interfere in IL-17 and IL-23 [138] signalling pathways, whereby microbiota modulates keratinocytes and immune cells [95]. Thus, probiotic administration in psoriasis improves the clinical manifestation of the disease. However, it is essential to note that probiotic treatment has yet to be standardised due to variations in probiotics content, the bacteria concentration employed and the concomitant prebiotic administration [139].

Both animal and human studies have demonstrated the effects of probiotics on improving psoriasis. In experimental model studies mainly involved imiquimod-induced psoriasis [140], probiotics were generally found to improve psoriasis-like characteristics and suppress pro-inflammatory cytokines IL-17 [141]. Some studies have associated psoriasis with T cell activation mediators, whereby probiotics help regulate T cells and decrease dryness and inflammation of the skin [142]. For instance, severe pustular psoriasis unresponsive to methotrexate, dapsone, and steroids has displayed significant clinical improvement after receiving *Lactobacillus sporogenes* supplements three times a day for two weeks with nearly absolute remission after four weeks [143]. Furthermore, in a study evaluating inflammatory biomarker and plasma cytokine levels in patients with ulcerative colitis, chronic fatigue syndrome and psoriasis in three randomised, double-blind, placebo-controlled trials, *B. infantis* administration for eight weeks was assessed. The investigators found a significant attenuation in TNF-α concentration compared to those treated with placebo [144]. Other human studies also observed probiotics’ effects on quality of life, reducing psoriasis severity, preventing relapses, improving mineral uptake in the gut and downregulation of pro-inflammatory markers [145].

Of particular interest is the interaction between polyphenol administration, the composition of the microbiota, and psoriasis development. Phenolic compounds have been studied for about two decades, playing a fundamental role in treating skin diseases such as psoriasis since they are associated with the inflammatory state [146]. Nonetheless, dietary polyphenols’ properties depend primarily on their bioavailability, which, in turn, is influenced by their polymerisation grade. The gut microbiota plays a crucial role in modulating the production, bioavailability, and, thus, the biological activities of phenolic metabolites, particularly after a food rich in polyphenols. In addition, evidence regarding the activity of dietary polyphenols on colonic microbial population composition or activity is recently emerging. However, although the impressive health-promoting activities of dietary polyphenols have been widely investigated, their effect on gut ecology modulation and its two-way relationship is still poorly understood. In this regard, in a study conducted by Medina et al., [147] from our research group to evaluate the effectiveness of extra virgin olive oil polyphenols in psoriasis treatment, 58 adult volunteers of both genders were assigned to one of two groups. Group 1 consisted of 24 patients with psoriasis who had not undergone any pharmacological treatment for at least one year, and Group 2 consisted of 34 patients with psoriasis receiving treatment for at least six months with steroids and/or another immunosuppressant. Both groups received the polyphenolic extracts prepared as a cream form twice daily for 16 weeks. Patients in group 2 continue the immunosuppressive treatment during the study. Polyphenols efficacy was evaluated by the Psoriasis Area and Severity Index (PASI) and the Dermatology Life Quality Index (DLQI). The investigators found at week 12 that 75% of patients in group 1 achieved a PASI 75 compared to 13% of patients in group 2 (*p* < 0.001), while at week 16, 100% of the patients in group 1 and 19% of patients in group 2 achieved a PASI 75 (*p* < 0.001).

Furthermore, at week 16, 59% of patients in group 2 reached PASI 50, which was also considered clinically significant. Regarding DLQI, 100% of group 1 patients were classified as responders at weeks 12 and 16 compared to 59% and 65% of group 2 patients. This study shows that polyphenolics isolated and purified from olive oil are effective and well-tolerated in treating psoriasis, especially if the patients were not primmed by immunosuppressant treatment. Similar findings have been found regarding polyphenols administration in actinic keratosis [148].

The current treatment of psoriasis in patients with obesity is directed towards a multidisciplinary approach [149,150] that contemplates a lifestyle change [151,152] through progressive weight loss, the combination of a diet rich in soluble fibre and physical exercise [153]. These changes modify the behaviour of adipocytokines that act on the immune-inflammatory state of psoriasis in patients with obesity [152], minimising the expression of the clinical manifestations of psoriasis that cause physical and psychological damage in this disease [150].

### 3.16. Antiobesity Therapy: Evidence for the Role of Bariatric Surgery and Incretin Analogues against Psoriasis

In recent years, it has been studied how emerging therapies with a high impact on the therapeutic approach to obesity, among which stand out: bariatric surgery (BS) and glucagon-like peptide-1 (GLP-1) analogues, can function in the improvement of the pro-inflammatory state of this disease and its systemic consequences. In this sense, it has been shown that, after BS, the inflammatory profile and the disproportionate inflammatory activity of T cells (CD4+ and CD8+), Th1/Th2, and B cells decreases significantly [154]; these changes can directly ameliorate low-grade systemic inflammation and, therefore, position BS as a tool for the treatment and prevention of psoriasis in obese patients. In fact, Maglio et al. conducted a study on 1991 patients undergoing BS and 2018 controls to evaluate the effect of BS on the incidence of psoriasis and psoriatic arthritis. After analysis of the results, the authors noted that BS was uniquely associated with a lower incidence of psoriasis (HR: 0.65; 95% CI: 0.47–0.89; *p* = 0.008), whereas a higher incidence of obesity was associated with a higher risk of psoriasis [155].

However, Egeberg et al. reported that patients undergoing BS, specifically gastric bypass, had a significantly lower risk and better prognosis of psoriasis and psoriatic arthritis [156]. Another study assessing the role of BS was in ten obese patients with psoriasis showed that, after the intervention, 70% of them remained in remission for six months, and it was also shown that three of the four patients who were previously taking systemic drugs for the treatment of psoriasis discontinued the medication [157]. It is worth mentioning that more clinical and preclinical research is needed to substantiate further the clinical and molecular implications of BS as a therapeutic tool against psoriasis.

On the other hand, GLP-1 analogues such as liraglutide and semaglutide have stood out as anti-obesity drugs with significant effects on weight loss and, in addition, exhibit anti-inflammatory properties [158,159,160]. Thus, GLP-1 analogues may ameliorate the low-grade systemic inflammation associated with obesity and thus contribute to the treatment of psoriasis. Thus, a meta-analysis of randomised clinical trials showed that diabetic patients with plaques who underwent treatment with liraglutide had a significantly lower psoriasis area and severity index (PASI) (SD: −4.332; 95% CI: −7.611 to −1.053; *p* = 0.01) [161]. Similarly, it has been described how liraglutide can improve the Dermatology Life Quality Index (DLQI) of diabetic patients with psoriasis and decrease the expression of IL-17, IL-23, and TNF-α, all pro-inflammatory cytokines involved in psoriasis and obesity pathogenesis [162,163]. Similarly, Costanzo et al. reported that semaglutide produced clinical improvement in psoriatic lesions since the PASI in diabetic patients with psoriasis decreased by 19%, and the DLQI increased considerably. Since the clinical studies evidencing the therapeutic role of GLP-1 analogues in psoriasis have been studied in diabetic patients, it is necessary to develop new clinical trials including obese patients as the study population [164].

## 4. Conclusions

The link between obesity and psoriasis is vital when evaluating patients who share these pathologies. This review described and analysed several studies on the interactions, similarities and differences, triggers and aggravating factors, and pharmacologic treatment implications between obesity and psoriasis (genetic, molecular, immunological, microbiome). In this sense, psoriasis is an immune-mediated chronic inflammatory disease amplified by obesity and where its cardio-immune-metabolic complications are worsened by adipose tissue dysfunction in the obese population. Therefore, by treating this dysfunction, the clinical manifestations of psoriasis improve; an integrative approach to the manifestation and course of these diseases can significantly aid in treating their severity and improving these patients’ health.

## Figures and Tables

**Table 1:** Summary of clinical evidence correlating the anthropometric index to psoriasis.

Author (REF).	Methodology	Relevant Results
Norden et al. [58]	A prospective cohort analysis which evaluated the incidence of psoriasis according to BMI, in a sample of more than 1.5 million patients in the United States, over a period between 1 January 2008, through 9 September 2019	The crude incidence of psoriasis per 10,000 person-years was 9.5% (95% CI, 9.1–10.0) in normal weight patients, 11.9 (95% CI, 11.4–12.4) in overweight, 14.2 (95% CI, 13.6–14.9) in obese class 1 patients, and 17.4 (95% CI, 16.6–18.2) among obese class 2/3 patients.
Setty et al. [62]	A prospective longitudinal 14 years study assessed the relationship between BMI, WC, HC, and psoriasis incidence in 78,626 nurses aged 25 to 42 years in the USA with a biannual follow-up.	A total of 892 new cases of psoriasis were collected; the incidence rate was 82 per 100,000 person-years. The multivariate RRs for psoriasis were 1.40 (95% CI, 1.13–1.73) with BMI 25.0–29.9; 1.48 (95% CI, 1.15–1.91) BMI 30.0–34.9; and 2.69 (95% CI, 2.12–3.40) for BMI 35.0 or greater (*p* < 0.001).
Castaldo et al. [48]	Open-label, single-arm, clinical trial, in 37 adult patients, overweight or obese, with stable chronic plaque psoriasis, without previous treatment. Patients underwent a WL program, through a protein-sparing VLCKD for four weeks and subsequently a Mediterranean-type, hypocaloric diet for six weeks.	The diet produced a reduction in the mean body weight of 12% (−10.6 kg), as well as a significant reduction in the mean PASI score of −10.6 (95% CI, −12.8 to −8.4; *p* < 0.001). 97% (n = 36) and 64.9% (n = 24) of patients recorded a PASI score response ≥50% and ≥75%, respectively. In addition, a reduction in the DLQI score of −13.4 points was observed.

Abbreviations: BMI: Body mass index; WC: Waist circumference; HC: Hip circumference; RR: Relative risk; WL: Weight loss; VLCKD: very low-calorie ketogenic diet; PASI: Psoriasis Area and Severity Index; DLQI: Dermatology Life Quality Index.

## Data Availability

Not applicable.
